# Subtractive genomics profiling for potential drug targets identification against *Moraxella catarrhalis*

**DOI:** 10.1371/journal.pone.0273252

**Published:** 2022-08-25

**Authors:** Bilal Ashraf, Nimrah Atiq, Kanwal Khan, Abdul Wadood, Reaz Uddin

**Affiliations:** 1 Baqai Institute of Information Technology, Baqai Medical University Karachi, Karachi, Pakistan; 2 Dr. Panjwani Center for Molecular Medicine and Drug Research, International Center for Chemical and Biological Sciences, University of Karachi, Karachi, Pakistan; 3 Hamdard Institute of Engineering Sciences and Technology, Hamdard University, Karachi, Pakistan; 4 Department of Biochemistry, Abdul Wali Khan University, Mardan, Pakistan; Instituto Butantan, BRAZIL

## Abstract

*Moraxella catarrhalis (M*. *catarrhalis*) is a gram-negative bacterium, responsible for major respiratory tract and middle ear infection in infants and adults. The recent emergence of the antibiotic resistance *M*. *catarrhalis* demands the prioritization of an effective drug target as a top priority. Fortunately, the failure of new drugs and host toxicity associated with traditional drug development approaches can be avoided by using an *in silico* subtractive genomics approach. In the current study, the advanced *in silico* genome subtraction approach was applied to identify potential and pathogen-specific drug targets against *M*. *catarrhalis*. We applied a series of subtraction methods from the whole genome of pathogen based on certain steps i.e. paralogous protein that have extensive homology with humans, essential, drug like, non-virulent, and resistant proteins. Only 38 potent drug targets were identified in this study. Eventually, one protein was identified as a potential new drug target and forwarded to the structure-based studies i.e. histidine kinase (UniProt ID: D5VAF6). Furthermore, virtual screening of 2000 compounds from the ZINC database was performed against the histidine kinase that resulted in the shortlisting of three compounds as the potential therapeutic candidates based on their binding energies and the properties exhibited using ADMET analysis. The identified protein gives a platform for the discovery of a lead drug candidate that may inhibit it and may help to eradicate the otitis media caused by drug-resistant *M*. *catarrhalis*. Nevertheless, the current study helped in creating a pipeline for drug target identification that may assist wet-lab research in the future.

## Introduction

*Moraxella (Branhamella) catarrhalis*, previously recognized as *Neisseria catarrhalis* or *Micrococcus catarrhalis* is a gram-negative and an aerobic diplococcus that is predominantly reported to be found in an upper respiratory tract commensal. *M*. *catarrhalis* has emerged as notorious bacterial pathogen in past 20 to 30 years [1]. It causes acute otitis media in infants and exacerbation of chronic bronchitis in adults. It is typically associated with various infections associated with other deadly pathogens like *Streptococcus pneumoniae* or *Haemophilus influenzae* being encountered in up to 50% of cultures [2]. *M*. *catarrhalis* is a prime cause of various active infections in hosts with weakened immune systems, encompassing pneumonia, endocarditis, septicemia, and meningitis [1]. Furthermore, hospital outbreaks of *M*. *catarrhalis*-related respiratory disease have been characterized and classified it as a nosocomial pathogen. For decades, *M*. *catarrhalis* was thought to be a harmless commensal since little is known about its pathogenic features and virulence factors despite the fact that research in this field has expanded in recent years [3].

The classical antibiotic treatment alleviates the clinical burden. However, unrestricted antibiotic use is a major factor in the rapid progress of antibiotic-resistant bacteria, which has reduced the number of viable antimicrobial options [4]. In recent past the antimicrobial resistance has been rising dramatically. Acute Chronic Obstructive Pulmonary Disease (COPD) and other respiratory diseases caused by *M*. *catarrhalis* are notoriously difficult to treat because of the rising MICs and antimicrobial drug resistance [5].

The genome and proteome analysis aids in the identification of several potential drug targets for the treatment of highly pathogenic diseases. Arguably comparative and subtractive genomic analysis is an exigent job because of its high dimensional data analysis. Interestingly, the arrival of the post-genomic era and pathogen whole-genome sequences opened multiple avenues for the methodologies such as comparative subtractive genomics to design new drugs and vaccine targets. This cost-effectiveness has unlocked the new pathways for finding potential drug candidates and it has accelerated the process of drug discovery, expanded the number of treatment options, and reduced the failure rate in clinical trial process in later stages [6]. Computational approaches enabled the identification of potent therapeutic targets against such pathogens [7]. The method has already been used to successfully prioritize and predict therapeutic targets for *Clostridium botulinum* [8], *Mycoplasma pneumoniae* [9], *Rickettsia* [10], *Neisseria gonorrhoeae* [11], *Salmonella typhi* [6], and *Shigella dysenteriae* [12].

In the present study, genomics data in BBH18 of *Moraxella catarrhalis* was investigated to find unique therapeutic targets and therapeutic candidates. The study includes comparative and subtractive genomics analysis approach, Protein-Protein Interaction (PPI) network analysis, essentiality, drug ability of target proteins and ADMET properties. Eventually, certain limitations from previous studies against *M*. *catarrhalis* such as consideration of hub nodes, and conserved drug targets are covered in this study. Future study may involve the development of antibacterial lead compounds against these shortlisted potential drug targets.

## Material and methods

**Fig 1:**
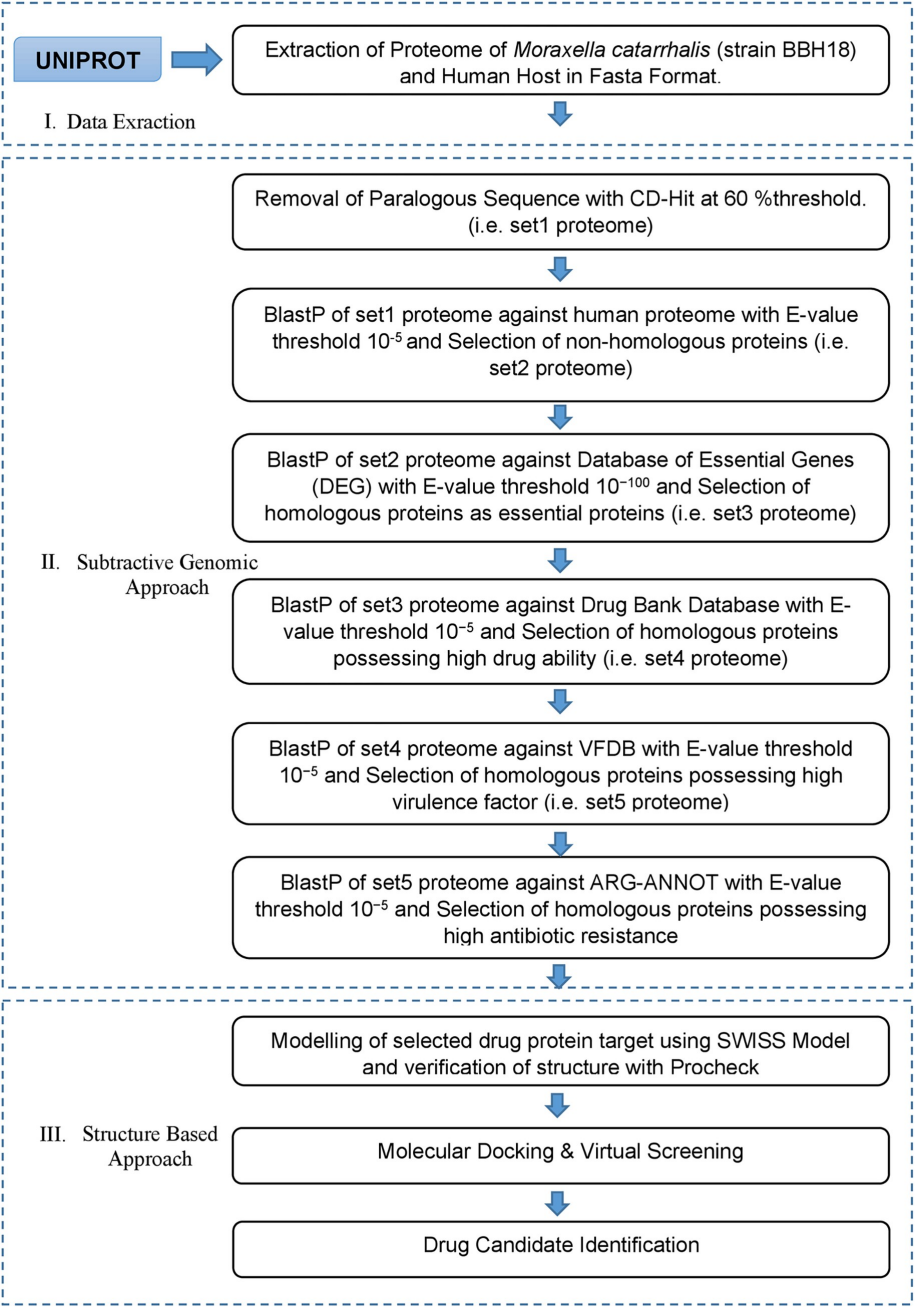
Flow chart: A general sketch of the current study integrated with the use of various computational approaches and tools to identify potential drug targets against *M*. *catarrhalis* BBH18.

Subtractive genomics approach was employed for the drug target prioritization against *M*. *catarrhalis* which holds the clinical and biological importance. The BBH18 strain was chosen to identify the potent drug target and candidate. There was not much reported work against this specific strain in the ground of *in silico* drug target identification and also it was the only reference strain available for *M*. *catarrhalis*, that’s why it was selected for further study. Several databases and tools as illustrated in the flow chart in Fig 1 were used for the determination of therapeutic targets.

### Retrieval of proteomes of pathogen and host

The whole proteome of *Moraxella catarrhalis* BBH18 and Human host both were obtained from the Universal Protein Resource (UniProt) database [13]. Additionally, the Database of Essential Genes (DEG database) [14, 15] was used to screen the drug targets essentiality, and the Drug Bank database Version 5.1.8 [16] was used to investigate the drug ability of proposed targets. Moreover, the Virulence Factor Database (VFDB) [17] was used to curate information about virulence factors of *M*. *catarrhalis* whereas, the ARG-ANNOT (Antibiotic Resistance Gene-ANNOTation) AA V6 July 2019 [18] was used for the detection of already existing and putative new Antibiotic Resistance (AR) genes in pathogen genomes [18].

### Subtractive genomics approach

Subtractive Genomics is an extensively employed approach that is used to subtract the sequences between the host and pathogen proteomes and metabolic pathways to provide details for a set of proteins that are required by the microorganism but do not exist in the corresponding host. Subtractive Genomics has an active role in identifying unique and essential potent drug targets for pathogen to survive without changing the systematic metabolic pathways of the hosts [19].

### Removal of paralogous protein sequences

The complete proteome of *Moraxella catarrhalis* BBH18 was eradicated at 60% threshold using Cluster Database at High Identity with Tolerance i.e. CD-HIT [20, 21].The proteins possessing the sequence identity greater than 60% are paralogous to each other. The complete sequences of paralogous i.e. duplicates were removed keeping the non-paralogous sequences only for the downstream analysis.

### Identification of non-homologous protein

Further, the set of proteins retrieved after removal of paralogous were subjected to BLASTp [22] with the expectation value (i.e. E-value) cut-off of 10^−5^ against *Homo sapiens* proteome [21]. The BLASTp generated results i.e. ’Hits Found’ (homologous sequences amid pathogen and the host) and ’No-Hits Found’ (Non-homologous sequences). The non-homologous sequences with no resemblance to the human host were opted for further analysis.

### Identification of essential non-homologous genes

Essential proteins of any organism are those proteins that possess a significant role in cellular metabolism [23]. Hence, BLASTp of non-homologue *M*. *catarrhalis* proteins was performed against DEG. Strictly, essential proteins were sorted out in *Moraxella catarrhalis* by keeping a threshold E-value of 10^−100^. To screen out essential genes, a minimum cut off score of 100 was set [21]. It resulted in the protein data set that were non-homologous as well as essential to *M*. *catarrhalis*.

### Druggability of essential proteins

Later, nonhomologous essential proteins were evaluated using BLASTp against Food and Drug Administration (FDA) approved proteins that served as therapeutic targets and obtained from the DrugBank. The evaluation was performed with E-value cut-off of 10^−5^ for the discovery of drug–target-like ability of identified essential proteins for the prioritization of novel and unique therapeutic targets [21].

### Identification of essential virulent proteins

Furthermore, the selected genes were subjected to BLASTp against the Virulence Factor Database by setting E-value cut-off to 10^−5^ for determining the proteins possessing the highest virulence factor [24].

### Resistance proteins analysis

For further structure-based studies, only those proteins that possess high antibiotic resistance were required. The BLASTp analysis was performed for essential virulent proteins against ARG-ANNOT by setting E-value cut-off to 10^−5^ [24]. The resultant data set was of homologous essential proteins possessing high antibiotic resistance and was opted for further structure-based studies.

### Identification of subcellular localizations

Every protein has a distinct function at a specified locality. These regions are crucial because proteins are distributed to specific regions in the cell once they are released. Failure of the proteins to move to their adequate location may cause a variety of disorders. Therefore, PSORTb version 3.0.2 [25] and CELLO2GO [26] were used to determine the subcellular localization of all essential, drug like, and nonhomologous proteins. The underlying principle behind Subcellular Localization (SCL) is to run a search of BLAST over all nonhomologous proteins required against the proteins with a specified subcellular location. These tools classified proteins into distinct types based on their cellular location: cytoplasm, cytoplasmic membrane, inner membrane, outer membrane and periplasmic membrane, extracellular region, and undetermined [6, 24].

### Structure prediction and homology modelling

The shortlisted proteins from the subtractive genomic approach were evaluated and searched for their structures in the Protein Data Bank (PDB). The BLASTp was used to find a suitable template for protein structure modelling. If there is a lack of 3-dimensional structure, the protein structure can be modelled using the Swiss Model–Homology Modeller [27]. In the lack of an experimentally determined crystal structure of the protein, the homology modelling is the most precise and efficient method for constructing protein structures i.e. 3D. It works by comparing the sequences of proteins in the Protein Data Bank [24].

### Validation of protein structure

The modelled structures were validated using various tools based on their respective principles to perform the docking experiment against shortlisted proteins. i.e. PROCHECK [28] to evaluate the stereochemistry composition of a protein structure by analyzing its residue-by-residue geometry as well as the entire structural geometries of the protein. The ERRAT i.e. empirical atom based analyzing tool [29], and PSIPRED were used to estimate the *β*-sheets, *α*-helices, and random coils (secondary structure) of shortlisted proteins i.e. sequence based validation [30]. On the other hand, PROSA web server [31] which is employed to validate the modelled protein structure against the available structures supplied from PDB on the basis of Z-score [32].

### Ligand and active site prediction

As the structure was modelled, it was required to find the active site over which the ligand could attach to perform its tasks and key roles. In the absence of any ligand in the active site, the ligand was predicted using the template protein of modelled structure obtained as a result of BLAST search. This active site can be chosen for the docking of ligand against respective proteins.

### Molecular docking studies

Molecular docking is a computational technique used to predict non covalent binding of a macromolecule i.e. protein (receptor) and a ligand, initiating with their unbounded structures acquired from homology modelling [33]. The most effective ligand in molecular docking has the lowest docking score for its target protein. Using standard docking parameters of 10 times Lamarckian GA settings resulting in 27,000 generations through AutoDock v4.2 [34]. In the docking experiment, the modelled protein function as the target and the identified compound acted as the ligand [35].

### Redocking and virtual screening for the identification of novel drug candidates

The docking parameters were validated first by re-docking an ADP co-crystal ligand discovered within the binding site of histidine kinase. For the molecular docking, the conventional docking protocol was used with AutoDock. The ligand was docked and implemented using 250 times Lamarckian GA settings, resulting in a maximum of 27,000 generations and 2,500,000 evaluations. [36]. The re-docking was performed to assess the performance of docking program for its capability of reproducing the same crystal conformation of the bound ligand [36].

Further on, virtual screening of 2000 compounds from the ZINC database [37] was performed against the histidine kinase i.e. subjected drug target protein to identify novel drug candidates using AutoDock Vina [38]. These compounds were selected based upon the range of their molecular weight from 150 to 350 Da (Dalton) as according to the Lipinski’s rule of 5, molecular weight should be >500 Da and as well as due to their easy availability from inhouse library (institute’s library). For the grid points, 72 on X-axis, 112 on Y-axis and 104 on Z axis were selected whereas the parameters for grid center were selected at 55.3, 30.378, and 26.716, respectively [38]. The AutoDock Vina PDBQT Split 1.1.1 [39] was used to split the prepared PDBQT library into the required file. Virtual screening was carried out using the default parameters applied for docking study.

### Post docking analysis

The Molecular Operating Environment (MOE 2019.01) [40] was used to assess the docked ligand–protein interaction and depict the ligand’s H–bonds and hydrophobic interactions with the docked protein inside a range of 5 Å. Whereas mmff94s force field was used for energy minimization.

### Physiochemical property profiling and toxicity predictions

The physio-chemical properties (i.e. ADME properties) of a ZINC products library was examined in order to determine the important characteristics and parameters that may have a role in influencing the bioactivities. Estimation of compound drug-likeness is component of the physio-chemical analysis (e.g., Lipinski’s rule, lead resemblance), molecular weight, compound interaction with biological environment (e.g., cell permeability, skin permeability, intestinal permeable), biopharmaceutical properties (i.e., pKa value, solubility, etc.), interaction with plasma proteins, and drug bioavailability. Moreover, the pkCSM [41] and SwissADME [42] tools were used to analyze the Absorption, Distribution of Drug, Metabolism, and Excretion (ADME) qualities as well as a number of factors related to the pharmacological action of the drug [43].

### Prediction of protein-protein interaction of identified drug target

Histidine kinase was the identified drug target protein. It was found to be essential and with cytoplasmic properties predicted through Database of Essential Genes (DEG) was evaluated for interactions with other proteins. The STRING Version 11.5 (Search Tool for the Retrieval of Interacting Genes/Proteins) [44] is a database containing protein interactions that include both verified and anticipated interactions. Interactions can be both direct (physical) and indirect (functional). The STRING integrates interaction data from these sources statistically for many species and transmits information among these organisms as required. The database currently comprised of 5,214,234 proteins from 1133 species [45]. It was subjected to determine whether the identified drug target can act as hub protein and to validate their functional interactions [46]. These PPIs are classified as hub proteins using node degrees and clustering coefficients. Medium confidence value i.e. 0.40 (by default setting) was set as the minimum required interaction score for the PPIs.

## Results

### Subtractive genomics approach

The current study is an application of an efficient subtractive genomics approach as exhibited in Fig 1. The Fig 1 depicts the complete series of steps as well as the tools and databases used for the identification of potent drug targets against *Moraxella catarrhalis* BBH18. Furthermore, the *in-silico* evaluation exhibited that the complete proteome of *Moraxella catarrhalis* BBH18 was comprised of a total of 1881 proteins. The step-wise filtering of the proteins during the current study was shown in Table 1.

**Table 1:** Proteins clamp down in current study: Subtractive filtering of proteins from *M*. *catarrhalis* BBH18.

S.no	Steps Involve in Current Study	*Moraxella catarrhalis* Proteins
1	Complete proteome of *M*. *catarrhalis BBH18*	1881
2	Number of proteins left after removal of paralogs sequences	1879
3	BLASTp of proteins against human host proteome (E value 10^−5^)	1360
4	BLASTp of non-homologous proteins against DEG (E value 10^−100^)	91
5	BLASTp of non-homologous essential proteins against DBD (E value 10^−5^)	38
6	BLASTp of non-homologous essential proteins against VFDB (E value 10^−5^)	14
7	BLASTp of non-homologous essential proteins against ARG-ANNOT (E value 10^−5^)	4

### Removal of paralogous protein sequences

The CD-HIT tool resulted in a total of two paralogous proteins among 1881 proteins. Subsequently, the remaining 1879 proteins were found as non-paralogous.

### Non-homologous proteins identification

Furthermore, these proteins were then subjected to BLASTp analysis against the human proteome to opt non-homologous proteins to Human proteome. By sorting out the BLASTp results, total of 519 proteins showed similarity with human proteins and these proteins were refrained for the downstream analysis as they may cause cross-reactivity and undesired toxicity in humans. Therefore, for further analysis, a total of 1360 non-homologous proteins were opted.

### Identification of essential non-homologous genes

Moreover, the BLASTp search was performed against the DEG that comprises of a collection of essential genes found in a wide variety of pathogenic and non-pathogenic organisms (both pro- and eukaryotes). A total of 91 proteins were identified as essential proteins required for the viability of *M*. *catarrhalis* and could be proposed as the potent drug targets.

### Drug ability of essential protein

Additionally, above 91 proteins were further subjected to the BLASTp analysis against Drug Bank Database. Only proteins with considerable similarities in sequence to FDA-approved therapeutic targets were chosen and the rest were omitted through dataset. The BLASTp alignment search was resulted in 38 druggable proteins of *M*. *catarrhalis*.

### Identification of essential virulent and antibiotic resistance protein

Further on, these 38 proteins were evaluated using BLASTp analysis against the Database of Virulence factor. Only 14 of which were classified as essential virulent proteins i.e. proteins with high virulence factor of *M*. *catarrhalis*. However, only four proteins were identified as antibiotic resistance out of 14 shortlisted proteins against ARG-ANNOT database.

### Subcellular localization prediction

**Fig 2:**
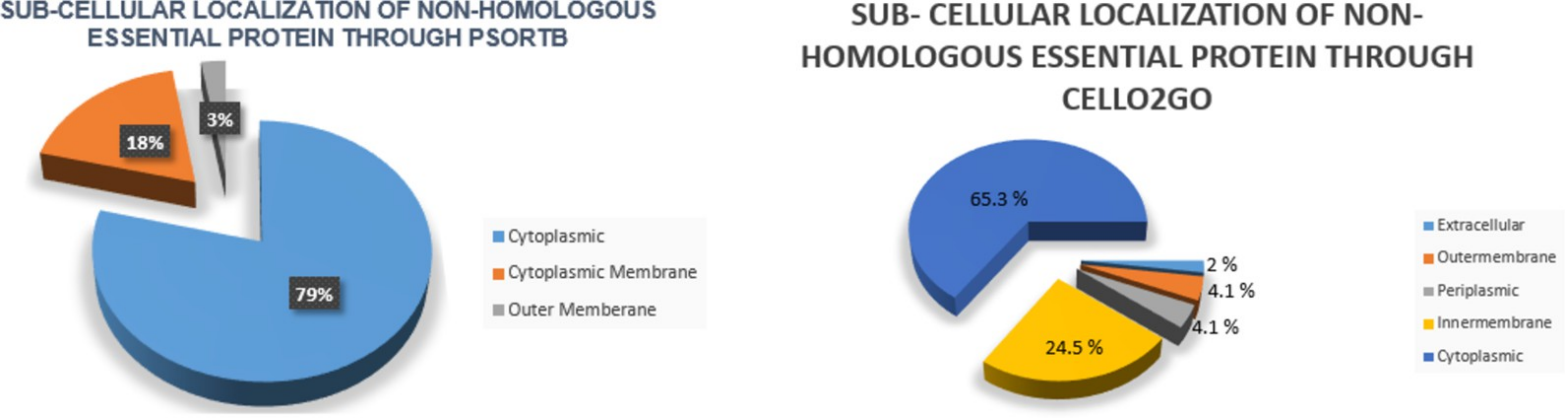
Subcellular localization: **(A)** Psortb results showing the subcellular distribution of 38 essential proteins identified in *M*. *catarrhalis* **(B)** CELLO2GO results showing the subcellular distribution of 38 essential proteins identified in *M*. *catarrhalis*.

In subtractive genomic approach, the PSORTb was employed to find the subcellular location of the nonhomologous essential proteins. In this research, among 38 essential drug-like proteins, 79% proteins were predicted to be found as cytoplasmic, 18% of them were anticipated to be in cytoplasmic membrane proteins, and only 3% were identified as outer membrane protein whereas according to CELLO2GO results, 65.3% proteins appeared to be cytoplasmic, 24.5% were shortlisted as the inner membrane proteins, 4.1% periplasmic proteins, 4.1% were classified as outer membrane proteins and 2% were found to be extra cellular proteins as shown in Table 2. The distribution of all essential proteins in *M*. *catarrhalis* was depicted in Fig 2A and 2B.

**Table 2:** Subcellular localization: Distribution of essential non-homologous proteins in a different area of cell.

S. No.	Uniprot Protein IDs	Protein’s Name	PSORTb Results	CELLO2 Results
1.	D5VBG0	Nitrate reductase (quinone)	**Cytoplasmic Membrane**	• Extracellular • Periplasmic • **Inner membrane**
2.	D5VA12	Anthranilate synthase component I	**Cytoplasmic**	• Inner membrane • **Cytoplasmic**
3.	D5VBG1	Nitrate reductase beta subunit NarH	**Cytoplasmic Membrane**	• **Inner membrane** • Cytoplasmic
4.	D5VBU9	Bifunctional chorismite mutase/prephenate dehydratase	**Cytoplasmic**	• **Cytoplasmic**
5.	D5VC88	LysR family transcriptional regulator	**Cytoplasmic**	• Inner membrane • **Cytoplasmic**
6.	D5VAI9	Biotin synthase	**Cytoplasmic**	• **Cytoplasmic**
7.	D5V959	Phosphomannomutase	**Cytoplasmic**	• Extracellular • **Cytoplasmic**
8.	D5VAP4	Efflux pump membrane transporter	**Cytoplasmic Membrane**	• **Inner membrane**
9.	D5VA96	Isocitrate dehydrogenase [NADP]	Cytoplasmic	• Periplasmic • **Cytoplasmic**
10.	D5V9R5	(P)ppGpp synthetase I SpoT/RelA	**Cytoplasmic**	• **Cytoplasmic**
11.	D5V9B6	Riboflavin biosynthesis protein RibD	**Cytoplasmic**	• **Cytoplasmic**
12.	D5V8W4	Histidinol dehydrogenase	**Cytoplasmic**	• **Cytoplasmic**
13.	D5V8D0	30S ribosomal protein S4	**Cytoplasmic**	• **Cytoplasmic**
14.	D5VCJ3	Uroporphyrinogen III methylase	**Cytoplasmic**	• Inner membrane • **Cytoplasmic**
15.	D5VB92	RNA polymerase sigma factor	**Cytoplasmic**	• **Cytoplasmic**
16.	D5VAG3	Aconitate hydratase B	**Cytoplasmic**	• **Cytoplasmic**
17.	D5VCG6	Phosphoenolpyruvate synthase	**Cytoplasmic**	• **Cytoplasmic**
18.	D5VD38	Dihydroorotase	**Cytoplasmic**	• **Cytoplasmic**
19.	D5VCW3	Dctp deaminase	**Cytoplasmic**	• **Cytoplasmic**
20.	D5VA77	Cell division protein FtsZ	**Cytoplasmic**	• Inner membrane • **Cytoplasmic**
21.	D5V8Z4	Peptidoglycan synthetase FtsI	**Cytoplasmic Membrane**	• Outer membrane • **Inner membrane**
22.	D5VDC9	Tryptophan synthase alpha chain	**Cytoplasmic**	• **Cytoplasmic**
23.	D5V8E7	30S ribosomal protein S3	**Cytoplasmic**	• Inner membrane • **Cytoplasmic**
24.	D5VCL6	Chorismate synthase	**Cytoplasmic**	• **Cytoplasmic**
25.	D5V9M8	Type IV pilus assembly ATPase PilB	**Cytoplasmic**	• Inner membrane • **Cytoplasmic**
26.	D5V9Z2	Anthranilate synthase component I	**Cytoplasmic**	• **Cytoplasmic**
27.	D5VDD0	Tryptophan synthase beta chain	**Cytoplasmic**	• **Cytoplasmic**
28.	D5VAV0	RNA polymerase sigma factor RpoH	**Cytoplasmic**	• **Cytoplasmic**
29.	D5V8I9	Malate synthase G	**Cytoplasmic**	• **Cytoplasmic**
30.	D5V8Z2	UDP-N-acetylmuramoyl-tripeptide–D-alanyl-D-alanine ligase	**Cytoplasmic**	• **Cytoplasmic**
31.	D5VBR4	Anthranilate phosphoribosyltransferase	**Cytoplasmic**	• **Cytoplasmic**
32.	D5VAF5	Histidine kinase	**Cytoplasmic Membrane**	• **Inner membrane**
33.	D5V8U6	Ppx/GppA phosphatase	**Cytoplasmic**	• Inner membrane • **Cytoplasmic**
34.	D5VAF6	Histidine kinase	**Cytoplasmic Membrane**	• **Inner membrane** • Cytoplasmic
35.	D5VCJ1	Sulfite reductase	**Cytoplasmic**	• **Cytoplasmic**
36.	D5VBA5	Transferrin binding protein A TbpA	**Outer Membrane**	• Extracellular • **Outer membrane**
37.	D5V8I2	Exopolyphosphatase	**Cytoplasmic**	• **Cytoplasmic**
38.	D5V9S5	Histidine kinase	**Cytoplasmic Membrane**	• **Inner membrane** • Cytoplasmic

### Novel drug targets prediction

**Fig 3:**
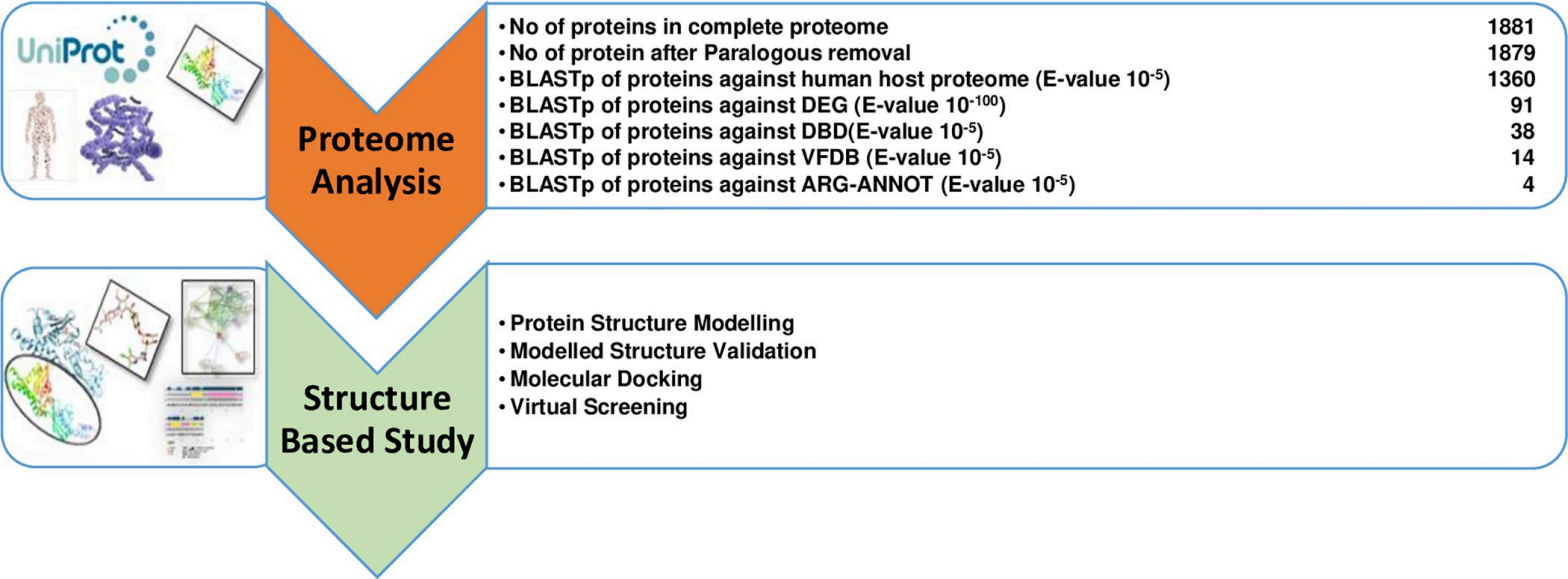
Current study summary for target protein identification: Stepwise analysis of subtractive genomic approach for drug targets identification in *M*. *catarrhalis*.

In this study, 38 potential drug targets were shortlisted as shown in Table 3. Because they are nonhomologous and non-paralogous, therefore these 38 proteins may be considered as promising therapeutic targets. Furthermore, among 38 potential drug targets, four of which were classified as antibiotic resistance proteins. Among them, one protein was shortlisted as essential, non-homolog, with high virulence factor and antibiotic resistance, drug able target against *M*. *catarrhalis* i.e. sensor histidine kinase (D5VAF6), and therefore, proceeded to structure-based studies. Fig 3 showed the comprehensive outcome of the current study.

**Table 3:** Non-homologous essential drug targets: 38 identified drug targets along with their proposed drug bank target ID.

S. No.	UNIPROT Protein IDs	Protein’s Name	Protein’s Function	Drug bank target Id
1.	D5VBG0	Nitrate reductase (quinone)	Supports anaerobic respiration on nitrate.	DB04464; DB07349
2.	D5VA12	Anthranilate synthase component I	Catalyzes the conversion of chorismite to anthranilate using ammonia as amino source.	DB01942
3.	D5VBG1	Nitrate reductase beta subunit NarH	Involves catalytic activity.	DB04464; DB07349
4.	D5VBU9	Bifunctional chorismite mutase/prephenate dehydratase	Responsible for synthesizing both phenylalanine and tyrosine.	DB08648
5.	D5VC88	LysR family transcriptional regulator	Function as either activators or repressors of gene expression.	DB03793
6.	D5VAI9	Biotin synthase	Catalyzes the final step in the biotin biosynthetic pathway, the conversion of dethiobiotin (DTB) to biotin.	DB03754; DB03775
7.	D5V959	Phosphomannomutase	Catalyzes the interconversion of mannose-6-phosphate and mannose-1-phosphate.	DB02007; DB02843; DB02867; DB02900; DB04522
8.	D5VAP4	Efflux pump membrane transporter	Extrudes a wide range of antibiotics.	DB03825; DB04209; DB07690
9.	D5VA96	Isocitrate dehydrogenase [NADP]	Catalyzes the oxidative decarboxylation of isocitrate to alpha-ketoglutarate.	DB01727; DB03461
10.	D5V9R5	(P)ppGpp synthetase I SpoT/RelA	Synthesizes and degrades (p)ppGpp.	DB02836; DB04315
11.	D5V9B6	Riboflavin biosynthesis protein RibD	Converts 2,5-diamino-6-(ribosylamino)-4(3h)-pyrimidinone 5’-phosphate into 5-amino-6-(ribosylamino)-2,4(1h,3h)-pyrimidinedione 5’-phosphate.	DB04280
12.	D5V8W4	Histidinol dehydrogenase	Catalyses the terminal step in the biosynthesis of histidine in bacteria, fungi, and plants, the four-electron oxidation of L-histidinol to histidine.	DB03811; DB04077; DB04447
13.	D5V8D0	30S ribosomal protein S4	Induces widespread conformational rearrangements in the 16S RNA.	DB00254; DB00256; DB00453; DB00595; DB00618; DB01017
14.	D5VCJ3	Uroporphyrinogen III methylase	Catalyzes the methylation reaction of CobA.	DB01752; DB04522
15.	D5VB92	RNA polymerase sigma factor	Responsible for determining the specificity of promoter DNA binding.	DB08874
16.	D5VAG3	Aconitate hydratase B	Catalyzes isomerization of citrate to isocitrate via cis-aconitate in the TCA cycle.	DB04351
17.	D5VCG6	Phosphoenolpyruvate synthase	Involved in glycolysis in the modified Embden-Meyerhof pathway in Thermococcus kodakarensis.	DB08357
18.	D5VD38	Dihydroorotase	Functions in the pathway for the biosynthesis of pyrimidine nucleotides by catalyzing the reversible interconversion of carbamoyl aspartate and dihydroorotate.	DB02129; DB02262; DB03801; DB04252
19.	D5VCW3	Dctp deaminase	Catalyzes the deamination of Dctp forming Dutp.	DB02333; DB03258
20.	D5VA77	Cell division protein FtsZ	Recruits other cell division proteins to the septum to produce a new cell wall between the dividing cells.	DB01864; DB04272;DB04315
21.	D5V8Z4	Peptidoglycan synthetase FtsI	Catalyzes the synthesis of cross-linked peptidoglycan from the lipid-linked precursors.	DB01147; DB01413
22.	D5VDC9	Tryptophan synthase alpha chain	Catalyzes the final steps in the biosynthesis of l-tryptophan from l-serine (Ser) and indole-3-glycerol phosphate (IGP).	DB03171; DB04272
23.	D5V8E7	30S ribosomal protein S3	Discriminates against aminoacyl transfer RNAs that do not match the codon of messenger RNA.	DB00759
24.	D5VCL6	Chorismate synthase	Catalyzes the last step in the common shikimate pathway leading to aromatic compounds such as the aromatic amino acids.	DB03247
25.	D5V9M8	Type IV pilus assembly ATPase PilB	Functions as a signaling protein to regulate exopolysaccharide production in Myxococcus xanthus.	DB04395
26.	D5V9Z2	Anthranilate synthase component I	Catalyzes the two-step biosynthesis of anthranilate.	DB01942
27.	D5VDD0	Tryptophan synthase beta chain	Catalyzes the biosynthesis of tryptophan from indol-3-glycerol phosphate and serine to tryptophan.	DB03171; DB04143; DB07732; DB07745; DB07748; DB07773; DB07890; DB07894; DB07925; DB07951; DB07952; DB07953
28.	D5VAV0	RNA polymerase sigma factor RpoH	Involved in regulation of expression of heat shock genes.	DB08874
29.	D5V8I9	Malate synthase G	Diverts carbon skeletons away from the catabolic reactions of the tricarboxylic acid cycle by providing a route from isocitrate directly to gluconeogenic precursors.	DB01992; DB03499; DB04343
30.	D5V8Z2	UDP-N-acetylmuramoyl-tripeptide–D-alanyl-D-alanine ligase	Involved in cell wall formation.	DB06970
31.	D5VBR4	Anthranilate phosphoribosyltransferase	Involved in tryptophan biosynthesis, catalyzing the transfer of a phosphoribosyl group to anthranilate, leading to the generation of phosphoribosyl anthranilate.	DB01632
32.	D5VAF5	Histidine kinase	Sense external environmental changes.	DB04395
33.	D5V8U6	Ppx/GppA phosphatase	Regulates stringent response in bacteria.	DB03382
34.	D5VAF6	Histidine kinase	Sense external environmental changes.	DB04395
35.	D5VCJ1	Sulfite reductase	Catalyzes the reduction of sulfite to hydrogen sulfide and water.	DB02832
36.	D5VBA5	Transferrin binding protein A TbpA	Removes iron and transport it into the periplasmic space.	DB02415; DB04147
37.	D5V8I2	Exopolyphosphatase	Involved in iron metabolism and is responsible for ferric-ion delivery.	DB03382
38.	D5V9S5	Histidine kinase	Sense external environmental changes.	DB04066

### Significance of selected protein

Sensor histidine kinase is an ATP-binding signal transduction protein found in *M*. *catarrhalis’s* two-component system [47]. These sensor histidine kinases detect changes in the environment (such as stress or the presence of a drug) surrounding the pathogen and transmit the signals inside that dynamically adjusts the internal mechanism of bacterial cells, preparing them to take advantage of these changes. Changes in these sensor kinases have been linked to resistance to many antibacterial drugs such as cefotaxime [48].

### Homology modeling of shortlisted drug target

**Fig 4:**
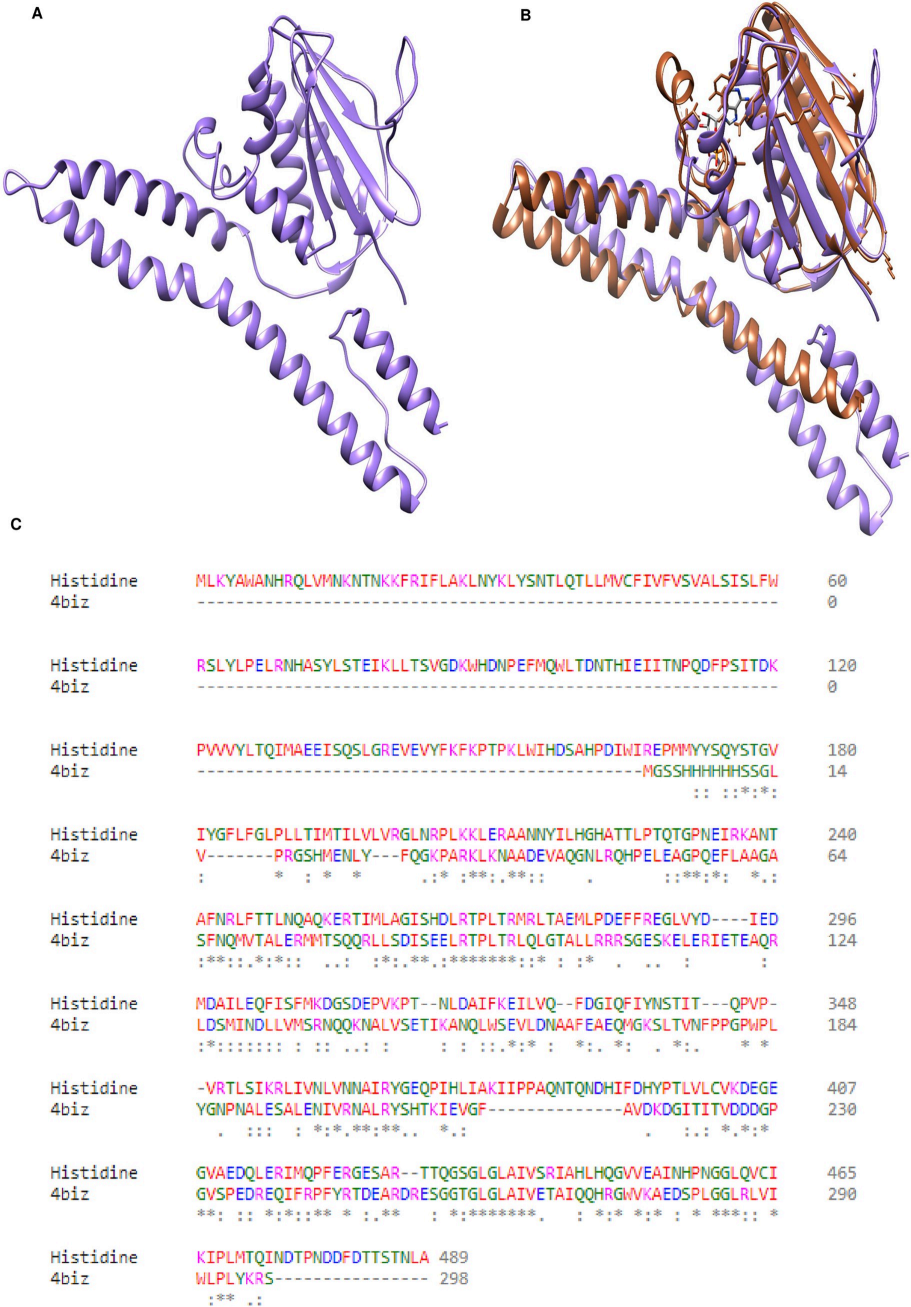
(A) Modeled Structure of Proteins (drug targets): Structure modeled through Homology SWISS modeler for sensor histidine kinases using 4biz as respective template. (B) Template Protein and Modelled Protein: Superimpose protein of histidine kinase with template protein in slenna color and modelled protein in medium purple. (C) Protein sequence alignment of modelled protein i.e. histidine kinase and template protein i.e. 4biz generated through clustal omega showing sequence similarity.

Histidine kinase’s 3-dimensional structure (one amongst four nominated and shortlisted) was not available in Protein Data Bank (PDB) PDB. As a result, the protein’s FASTA sequence from the NCBI database possessing the accession number D5VAF6 as specified in the database was used for the homology modelling. 4CTI, 4BIU, and 4BIZ were the respective structures of PDB that could be possible templates with percent identities of 34.67%, 27%, and 26.69%, respectively. Ultimately, the structure 4BIZ with a 26.69% sequence similarity and 54% query coverage was selected as a template due to its similarity and availability of ligand, and the structure was effectively modelled as shown in Fig 4.

### Modelled structure’s validation

Various tools were used to verify the modelled protein structure, i.e.

*I) Confirmation of Proteins through PSIPRED*
  The PSIPRED was used for the secondary structure validation of the protein. It validated the structure on the prediction of helices and beta sheets formation as shown in S1 Fig
*II) PROCHECK Validation of Proteins*
  The PROCHECK was used to generate a Ramachandran plot for the modelled protein structure. The Ramachandran plot showed about 91.0% residues found in the favorable region, having one residue in the disallowed region, 16 residues in the additionally allowed regions whereas 4 in generously allowed regions responsible for about 6.8% and 1.7%, correspondingly as shown in S2 Fig.
*III) ERRAT Validation of Proteins*
  The ERRAT tool was used to validate the unbounded statistics between two atoms conformation in the structure. It resulted in the quality factor of about 89.147% as shown in S3 Fig.
*IV) PROSA web Validation of Proteins*
  The ProSa web server tool was used to calculate the quality of the 3-D structures of proteins in terms of Z-score in the structure of modelled protein. The resulted Z-score is -7.33. The Z-score was calculated using NMR Spectroscopy (dark blue) and X-ray crystallography (light blue) in relation to length of protein chains in the provided structure as shown in S4A Fig. The energy plot illustrates the local model’s quality by depicting the knowledge-based energies as a function of amino acid sequence location as shown in S4B Fig.

### Prediction of active site

**Fig 5:**
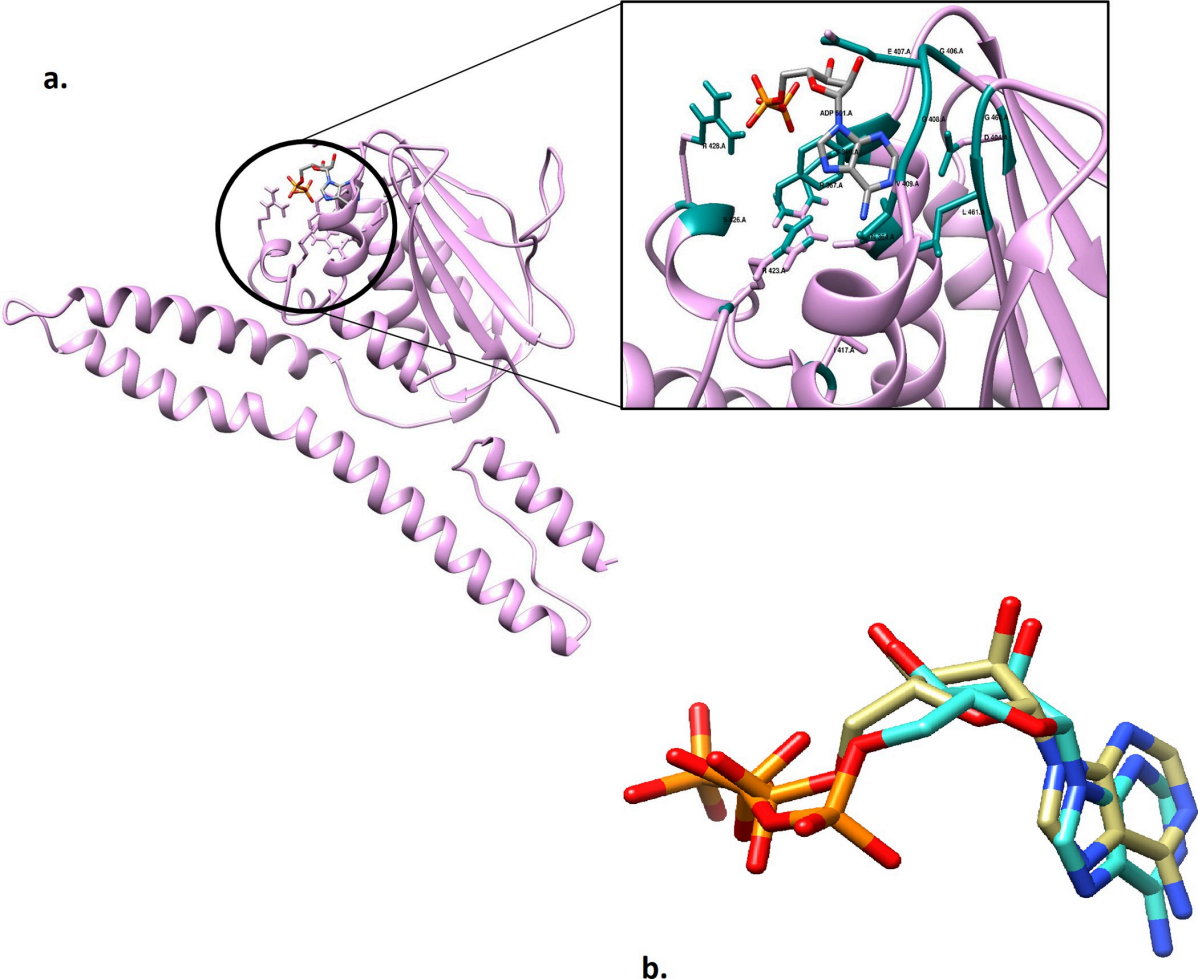
A. Predicted Active Site for histidine kinase: i.e. depicted by R423 (1.330 Å), L461 (1.550 Å), E407 (1.546 Å), R428 (1.339 Å) and I417 (1.548 Å) residues. B. Pre-Docked and Post Docked Protein: the superimposed docked complex of pre docked ADP (in dark khaki) over post docked (in aqua) highlighting the accuracy of docking study in terms of RMSD indicated as 1.660 Å.

The active site of a protein can be predicted by a variety of bioinformatics tools including molecular docking assays and structure-based drug discovery. The anticipation of active site was performed on the basis of following amino acids and their respective binding energies as amino acid Arginine (R) is present at 423 position attached with binding energy of 1.330 Å, Leucine (L) is present at 461 position attached with binding energy of 1.550 Å, GlutamicIid (E) is present at 407 position attached with binding energy of 1.546 Å, Arginine (R) is present at 428 position attached with binding energy of 1.339 Å, and Isoleucine (I) is present at 417 position attached with binding energy of 1.548 Å as shown in Fig 5A.

### Protein-ligand interactions study through docking

The protein-ligand interactions were analyzed through AutoDock Vina.

*i) Ligand Identification*
  The ligand co-crystalized within the binding cavity of the template protein (i.e., ADP) was retrieved and shown in S5 Fig. This compound corresponds to the purine ribonucleoside 3’, 5’-bisphosphates class of organic compounds. These are purine ribonucleotides possessing one phosphate group connected to the ribose moiety such as 3’ and 5’ hydroxyl groups [16]. The IUPAC name of ADP is: {[(2R,3S,4R,5R)-5-(6-amino-9H-purin-9-yl)-4-hydroxy-3-(phosphonooxy)oxolan-2-yl]methoxy}phosphonic acid ligand was identified from a template protein PDB ID: 4BIZ (from *Escherichia coli*) for sensor histidine kinase.
*ii) Molecular Docking with AutoDock*
  The AutoDock 4.2 was used for the docking study of histidine kinase. By selecting 10 algorithms run along with setting the Lamarckian GA to 10 times, the ADP ligand was docked and a maximum number of evaluation steps of 2,500,000 proceeding the generation of 27,000. Binding of ligand in the active site of protein in various orientations and conformations was revealed because of AutoDock. Each conformation has a distinct binding energy, ranging from negative to positive. The lowest binding energy of -6.3 kcal/mol was aided in ranking the top conformation of ADP, since the lowest binding energy relates the ligand’s spontaneous binding to the active site, and also forms a lower energy complex which is more stable.

### Redocking and virtual screening for identification of novel drug candidates

The redocking validation of co-crystallized ligand yielded a binding energy prediction of -5.32 kcal/mol. Fig 5B depicts the top-ranked docked conformation of ADP with histidine kinase protein. The RMSD of the top-ranked docked ADP conformation against the co-crystal structure was 1.660 Å indicating that the docking parameters could be implemented for virtual screening.

**Fig 6:**
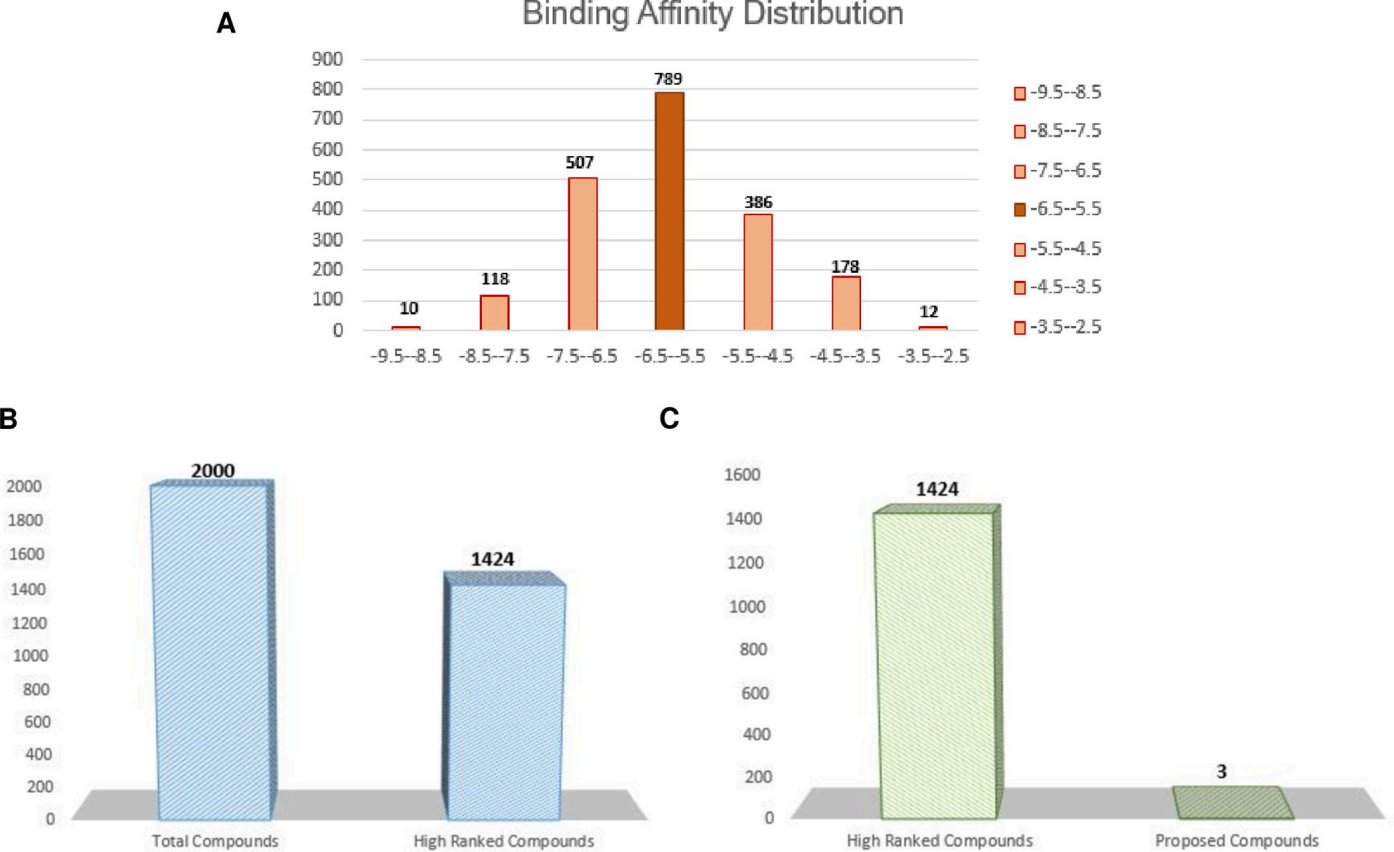
**(A)** Virtual screening of 2000 compounds, **(B)** identified leads like compounds, **(C)** and proposed leads compounds in current study.

The modelled structure was docked with the ZINC library. The compounds were screened using the same parameters that have previously been used for AutoDock validation (redocking). As seen in Fig 6A, the screening led to the identification of 789 compounds known as highly ranked with binding energies ranging from -5.5 to -6.5 kcal/mol. Whereas only 1424 molecules exhibited favorable interactions with histidine kinase with energetics spanning between -5.5 to -9.3 kcal/mol, as illustrated in Fig 6B. These compounds may serve as leads in the future. Furthermore, Fig 6C showed three potent drug candidates that are identified for histidine kinase against 2000 compounds from ZINC. These identified potent drug candidates are ZINC09185674, ZINC03839141, and ZINC00631248 possessing the binding energies as -6.4, -6.2, and -6.2 kcal/mol, respectively.

### Post docking analysis (i.e. interaction analysis of selected compounds with histidine kinase)

The post docking interaction analysis of shortlisted compounds was conducted to comprehend the identified mechanism of binding and pharmacological activity against histidine kinase. The rank order of docking depending on score and presented as following:

ZINC09185674 >ZINC03839141 and ZINC00631248 possessing the binding energies as -6.4, -6.2, and -6.2 Kcal/mol, respectively.

The docking analysis for ZINC09185674 revealed considerable binding energy of −6.4 kcal/mol. The ZINC09185674 was found to facilitate hydrophobic interactions only within binding cavity of histidine kinase. It mediates H–bonds as a hydrogen acceptor to Arg356 and Lys355 via the hydroxyl group and one H– bond as a hydrogen acceptor to Gln331 via the pyrrole ring’s oxygen.

The binding score of ZINC03839141 was -6.2 Kcal/mol. The hydrophobic interactions within the binding cavity are depicted. As a hydrogen acceptor, it bridges two hydrogen bonds with Tyr339. The ZINC03839141 was also interacted ionically with Asp332.

**Fig 7:**
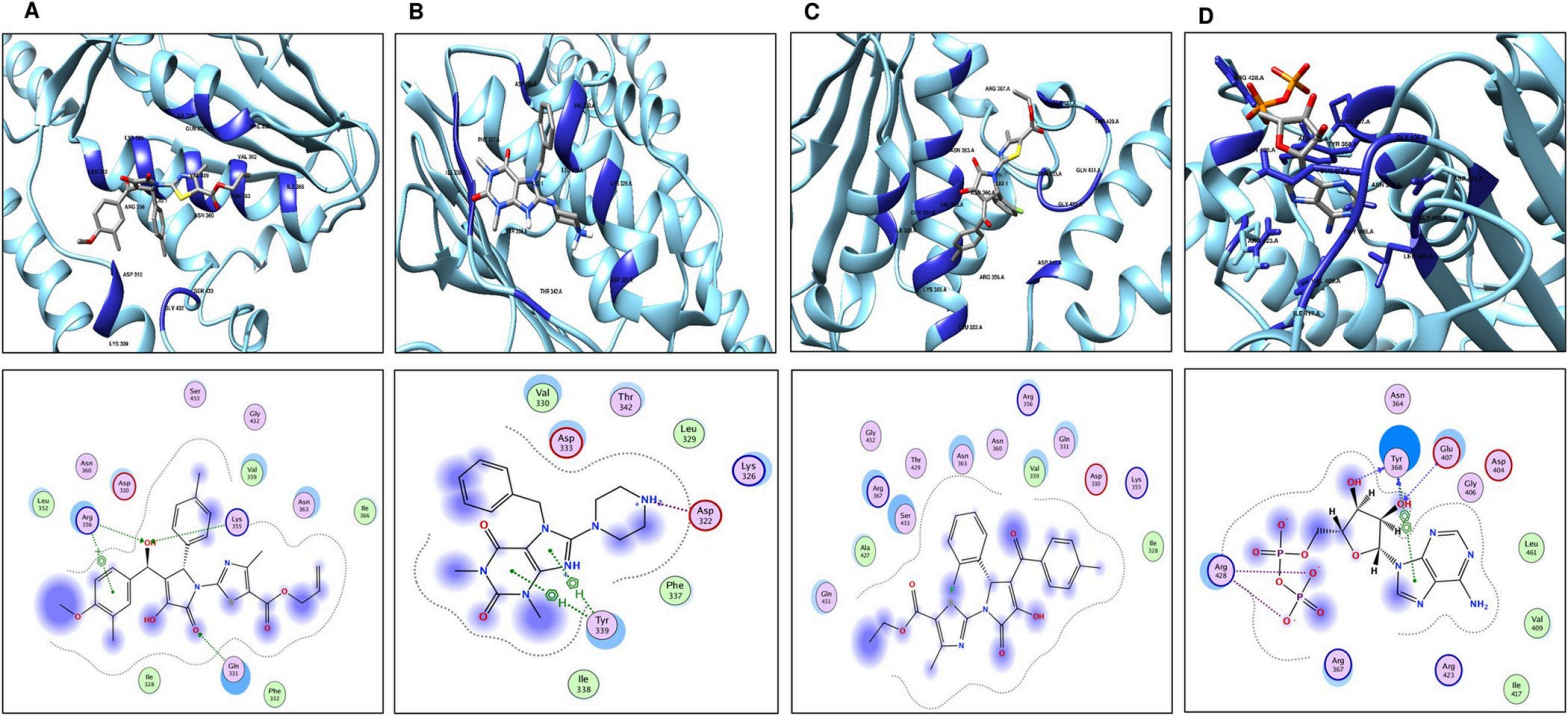
Redocked compound incorporating the modelled protein: **(A)** For ZINC09185674, **(B)** ZINC03839141, **(C)** ZINC00631248 and **(D)** Reference Protein.

With docking scores of 6.2 kcal/mol, ZINC00631248 has demonstrated strong hydrophobic interactions. Importantly, the reference compound (ADP) showed an ionic interaction with Arg428. It serves as a hydrogen donor Tyr368 and a hydrogen acceptor Glu407 in two H-bonds. through hydroxyl group that shows the pi-pi interaction. The redocked compound incorporating the modelled protein (2D and 3D interaction) is depicted in Fig 7. Table 4 showed the docked scores and reported types of bonds anticipated by the MOE tool for the identified compounds.

**Table 4:** Docking scores and identified bond types predicted through MOE tool for shortlisted compounds.

S. No.	Ligand	Receptor	Interaction	Distance	E (kcal/mol)	Binding Energy (kcal/mol)
1	**ZINC09185674**					
	O6	NE2 GLN331	H-acceptor	3.16	-1.2	-6.4
	O28	CE LYS355	H-acceptor	3.95	-0.6	
	O28	CG ARG356	H-acceptor	3.37	-0.5	
	6-ring	NH2 ARG356	pi-cation	3.60	-0.7	
2	**ZINC03839141**					
	C 1	O TYR339	H-donor	3.60	-0.6	-6.2
	N18	OD1 ASP322	ionic	3.16	-3.5	
	6-ring	N TYR339	pi-H	4.36	-2.5	
	6-ring	CB TYR339	pi-H	3.56	-1.2	
	5-ring	CD2 TYR 339	pi-H	3.79	-1.6	
3	**ZINC00631248** Hydrophobic interactions					-6.2
4	**Reference**					
	O3’ 14	O TYR 368	H-donor	3.50	-0.’	-6.32
	O2’ 16	O TYR 368	H-donor	3.08	-1.1	
	O1A 7	NH1 ARG 428	H-acceptor	3.13	-2.3	
	O1A7	NH2 ARG 428	H-acceptor	2.75	-8.2	
	O2A 8	NH1 ARG 428	H-acceptor	3.0’	-6.8	
	O2’ 16	N GLU 407	H-acceptor	2.94	-2.9	
	O2B 3	NE ARG 428	ionic	3.70	-1.2	
	O2B 3	NH1 ARG 428	ionic	2.91	-5.1	
	O2B 3	NH2 ARG 428	ionic	2.74	-6.5	
	O3B 4	NH2 ARG 428	ionic	3.91	-0.7	
	O1A 7	NH1 ARG 428	ionic	3.13	-3.7	
	O1A 7	NH2 ARG 428	ionic	2.75	-6.4	
	O2A 8	NH1 ARG 428	ionic	3.01	-4.4	
	5-ring	6-ring TYR 368	pi-pi	3.84	-0.0	

### Physiochemical property profiling and toxicity predictions

The pharmacokinetic parameters of three chosen drugs were calculated using the online pkCSM tool based on Blood-Brain Barrier crossing capabilities, drug-likeness, toxicological analyses and ADME characteristics. The Lipinski rule of five was employed in the drug-likeness characterization.

To anticipate the compound’s drug likeness, the SwissADME tool was employed. The two of three selected candidates have indicated zero violations to Lipinski’s Rule of Five whereas one compound has indicated only one violation and showed acceptable drug-like properties. The results of ADME properties analysis including Water Solubility, Blood-Brain Barrier (BBB) Permeability, Human Intestinal Absorption (HIA), Skin Permeability, CaCo2 permeability, and Lipinski Violation of shortlisted three compounds are shown in Table 5.

**Table 5:** ADMET Profiling: Properties analysis of shortlisted 3 compounds.

Proposed Potent Drug Candidate	Water Solubility	CaCo2 permeability	HIA	Skin Permeability	BBB Permeability	Lipinski Violation	Biochemical PAINS alert
ZINC09185674	-4.692	0.802	91.338	-2.735	No	Yes (1 violation)	0 alert
ZINC03839141	-2.709	1.453	70.453	-2.735	No	Yes (0 violation)	0 alert
ZINC00631248	-5.077	0.94	98.9	-2.735	No	Yes (0 violation)	0 alert

The results of toxicity analysis i.e. Max Tolerated Dose (Human), Minnow toxicity, Skin Sensitization, Hepatotoxic, Ames test, Oral Rat Acute Toxicity (LD50), *T*. *Pyriformis* (Toxicity) are shown in Table 6. The table also includes Radar of the respective compound.

**Table 6:** Toxicity analysis: Assessment of toxicity of shortlisted 3 compounds.

Proposed Potent Drug Candidate	Max Tolerated Dose (Human)	Minnow toxicity	*T*. *Pyriformis* (Toxicity)	Oral Rat Acute Toxicity (LD50)	Ames Test	Hepatotoxic	Skin Sensitization
ZINC09185674	0.33	-3.14	0.285	2.588	No	Yes	No
ZINC03839141	1.303	2.81	0.285	2.202	Yes	Yes	No
ZINC00631248	0.412	-2.289	0.286	2.496	No	Yes	No

### Prediction of protein-protein interaction of identified drug target protein

For the filtration and analysis of functional genomic data to annotate structural, functional, as well as evolutionary information on proteins, the proposed interaction could be utilized.

**Fig 8:**
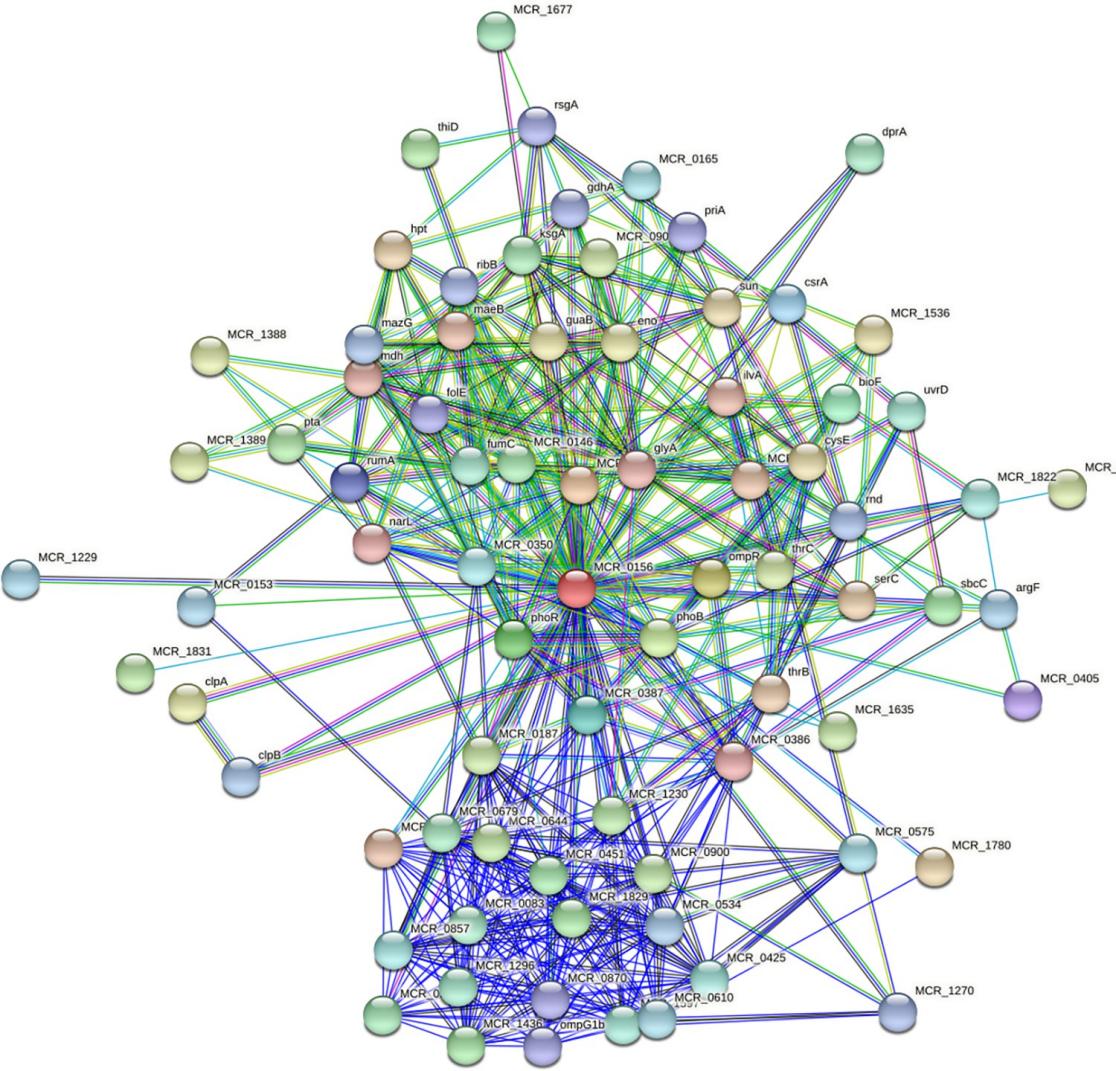
Protein-protein interactions: Schematic PPI network generated through the STRING database for histidine kinase.

Histidine kinase’s NCBI ID: D5VAF6 was submitted to the STRING database and found the interaction with other proteins in the *Moraxella catarrhalis*. The MCR _0156 represented the histidine kinase, and its minimal interactions with other proteins in their surroundings MCR_0386 (Two-component system sensor histidine kinase) with score of 0.721, MCR_0387 (Two-component system sensor histidine kinase) with score of 0.811, MCR_0405 (Tetratricopeptide repeat family protein) with score of 0.746, MCR_1062 (LuxR family transcriptional regulator) with score of 0.998, bioF (8-amino-7-oxononanoate synthase) with score of 0.844, csrA (Translational regulator CsrA) with score of 0.770, ompR (Two-component system response regulator) with score of 0.946, phoB (Two-component system phosphate regulon response regulator PhoB) with score of 0.896, phoR (Two-component system phosphate regulon sensor histidine kinase PhoR) with score of 0.894, anD RUmA (23S rRNA (uracil(1939)-C(5))-methyltransferase RlmD) with score of 0.760. The results showed that the histidine kinase (MCR_0156) protein has 482 edges, total 309 edges were shown to be expected, whereas number of nodes present are 78, and 12.4 is suggested as its average nodes degree. The PPI enrichment *p*-value is < 1.0e-16 with a local clustering score of 0.657 on average (Fig 8). These proteins are engaged in a variety of critical functions. Because of targeting Histidine kinase protein, the function of the other interacting proteins may be jeopardized. As a result, this protein might be used as a therapeutic target.

## Discussion

In present day and age, the computational methods and approaches have gained considerable attention for the identification and development of potent drug targets [49]. Yet, high-throughput sequencing experimental data for the majority of infectious bacteria is currently unavailable, and efforts to define and identify essential drug targets have now being relied solely on bioinformatics predictions [50]. Because of the obvious rise in drug resistance among pathogens, *in-silico* subtractive genomic analysis has been widely used for strain-specific targeting for drug target identification [51].

In the current research, subtractive genomics approach was employed for the identification and prediction of potent drug candidates. The focus of the current research was one of the most clinically relevant species i.e. *Moraxella catarrhalis* BBH-18 strain. It is one of the widely used approach for the novel drug targets identification against various deadly pathogens. The respective approach led to the identification of a total of 91 non-homologous, 38 essential druggable proteins, and 14 virulent protein of *M*. *catarrhalis*, out of which four were reported as potent drug targets. As a result, i.e., Efflux pump membrane transporter (D5VAP4), Histidine kinases (D5VAF5), (D5VAF6), and (D5V9S5) were identified as potent drug targets. These proteins may result in the removal and destruction of pathogen from the host through effective drug candidates and vaccines. Finally, one enzymatic protein was opted as potential drug target against *M*. *catarrhalis* i.e., sensor histidine kinases (D5VAF6) involved in two-component system. It plays the key role for the bacteria’s development and survival [52].

Histidine kinase has been reported in different studies as a potential drug target against various pathogens such as *Mycobacterium tuberculosis* [53], *Salmonella enterica* [54], *Streptococcus* Species [55], *Bacillus subtilis* and *Staphylococcus aureus* [53] and etc. But this study has uniquely reported histidine kinase for *M*. *catarrhalis* BBH18 as it was not documented as a drug target yet. Histidine kinase plays a major role in the two-component system of *M*. *catarrhalis*. It usually uses two-component signal transduction systems to translate extraneous and cellular signals into cell signaling. Because of the relevance of this protein’s function, it might be used as a potential therapeutic target in future [56].

Furthermore, ZINC library (>2000 compounds) was screened against the selected drug target to identify potential inhibitor. Following the screening process, 789 compounds having binding energies between -5.5 and -6.5 kcal/mol were identified as promising candidates. Only 1424 molecules showed preferential interactions with histidine kinase (energetics -5.5 to -9.3 kcal/mol). ADMET profiling was performed to substantially docked compounds to predict highly potent drug-like molecules. Subsequently, only three compounds were shortlisted as novel drug candidates that are ZINC09185674, ZINC03839141, and ZINC00631248. The binding energies of ZINC09185674, ZINC03839141, and ZINC00631248 are in descending order from lowest to highest, -6.4, -6.2, and -6.2 kcal/mol. To verify our docking analysis, molecular re-docking was performed for reference compound (ADP) using the same applied parameters. The results revealed the RMSD of 1.660 Å for redocking analysis validating the applied protocol of screening.

Bioinformatics subtractive genomics analysis is used to identify prospective therapeutic targets and candidates in this research. Genome and proteome pipelines are examined to prioritize effective antimicrobial agents that may be useful in halting the progression of the severe disease ‘Campylobacteriosis” followed by experimental verification. This might aid in the treatment of periodontal or other *C*. *concisus*-related disorders, as well as the reversal of *C*. *concisus*-induced intestinal microbial imbalance infections. The method employed has the potential to be used as a general method for target identification, and hence may be used in drug development.

The preceding research identified different essential proteins that could be used as potential drug targets and candidates. Concurrently, in this work, cytoplasmic protein can be typically utilized to identify drug targets, whereas membrane-associated proteins can be employed for formulation of peptide vaccines [57]. As a result, different other computational methodologies and approaches in addition to this approach and experimental validations can be used in the future to develop potential therapeutic strategies not only against *M*. *catarrhalis* but also against other pathogens.

## Conclusion

Notably, the analysis of genomes and proteomes of many pathogens has revolutionized the identification of therapeutic targets against pathogens. In this research, a subtractive genomic approach was employed to reveal beneficial findings in determining non-homologous essential druggable proteins against *M*. *catarrhalis*. These potential drug targets may aid in developing the novel antibiotics as well as potential drug targets that may be directed against *M*. *catarrhalis*, ensuring that the revealed targets are not the same as the host genome i.e. *Homo sapiens* in this case, to avoid any allergic responses or harmful consequences. By targeting these proteins functioning with novel drugs, candidates may be capable of damaging and eliminating infections from their respective hosts. The findings encompass all essential and potent drug targets in *M*. *catarrhalis* which could help future researchers to develop effective drug agents and vaccines against strain-specific *M*. *catarrhalis* BBH18.

## Supporting information

S1 Fig Secondary structure validation through PSIPRED predicts the positions for helices and beta sheets for histidine kinase. (TIF)

S2 Fig Ramachandran Plot generated through PROCHECK shows 91% residues in the favored region for histidine kinase. (TIF)

S3 Fig ERRAT tool for unbounded statistics between two atoms confirmation in the structures shows the quality factor of about 89.147%. (TIF)

S4 Fig ProSa web server tool calculates the quality of the 3-D structures of proteins in terms of Z-score i.e., -7.33 in the structure of modelled protein. (TIF)

S5 Fig Identified Ligands: ligand identified for drug targets, Adenosine Diphosphate(ADP), IUPAC names as {[(2R,3S,4R,5R)-5-(6-amino-9H-purin-9-yl)-4-hydroxy-3-(phosphonooxy)oxolan-2-yl]methoxy}phosphonic acid as ligand identified against histidine kinase protein. (TIF)

S1 Graphical abstract (TIF)
