# Supplementary figures and images for: Subtractive genomics profiling for potential drug targets identification against Moraxella catarrhalis
Source: PLoS One. 2022 Aug 25;17(8):e0273252. doi: 10.1371/journal.pone.0273252 (PMC9409589; doi:10.1371/journal.pone.0273252)

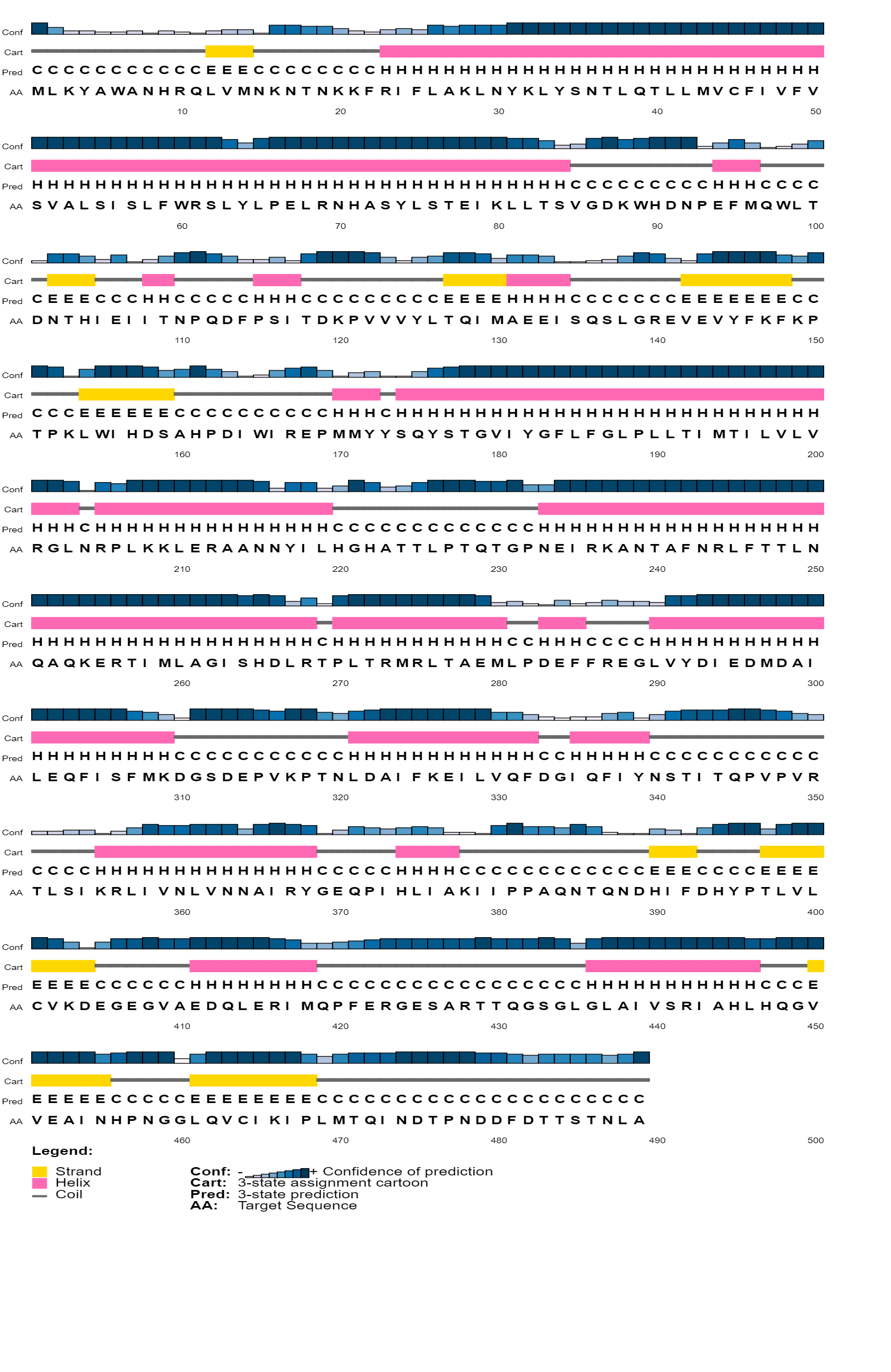

Supplement: S1 Fig — (TIF) [file pone.0273252.s001.tif]

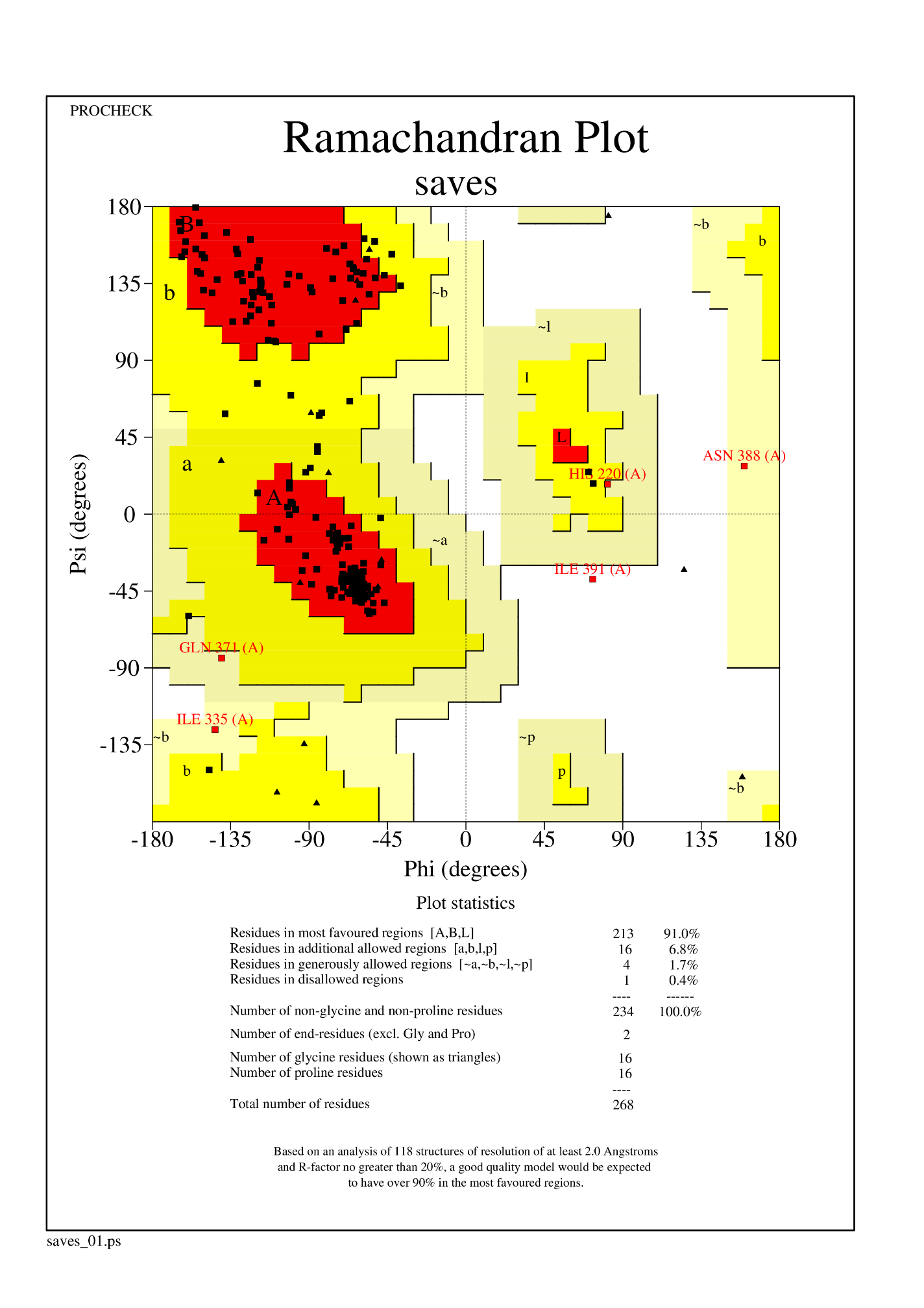

Supplement: S2 Fig — (TIF) [file pone.0273252.s002.tif]

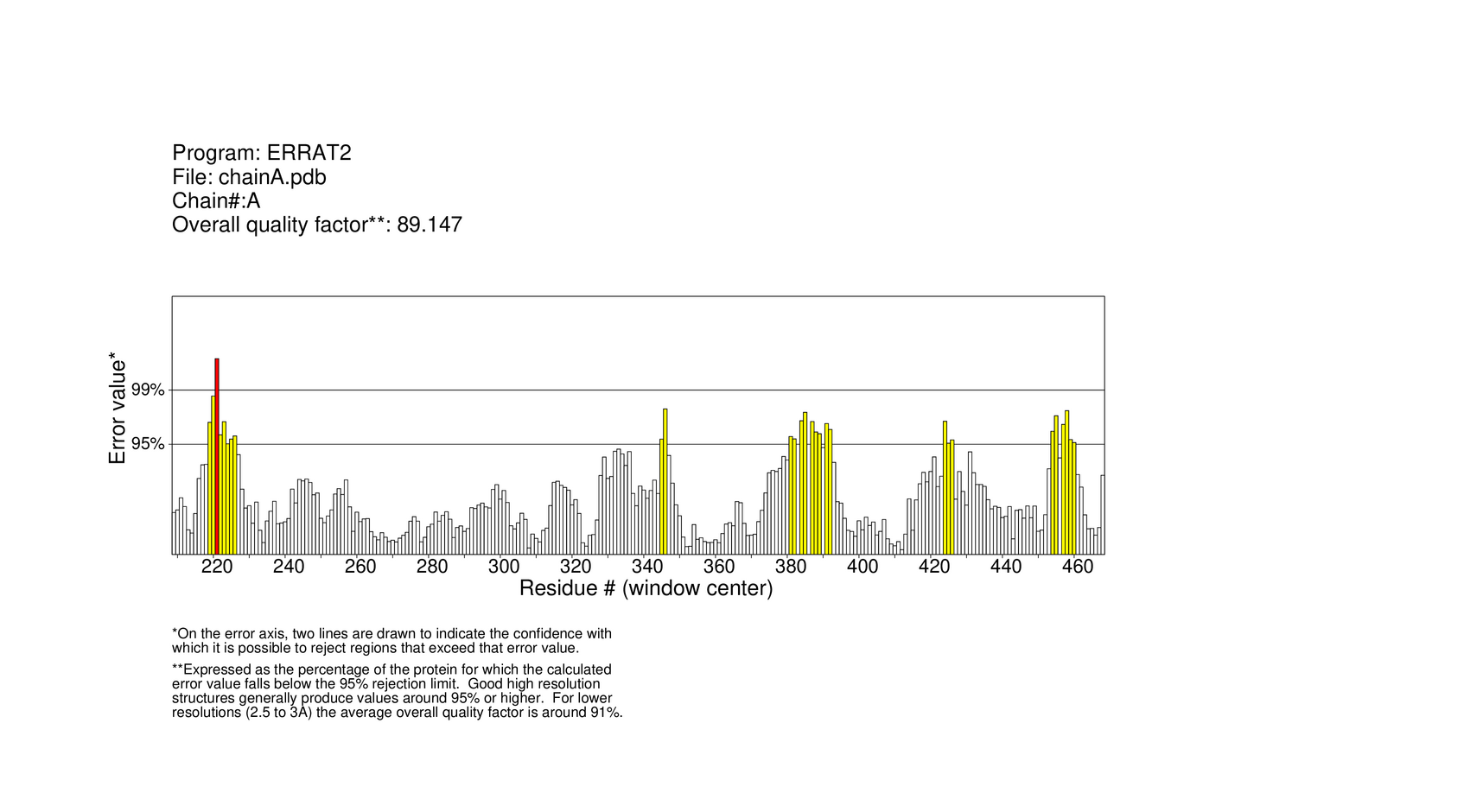

Supplement: S3 Fig — (TIF) [file pone.0273252.s003.tif]

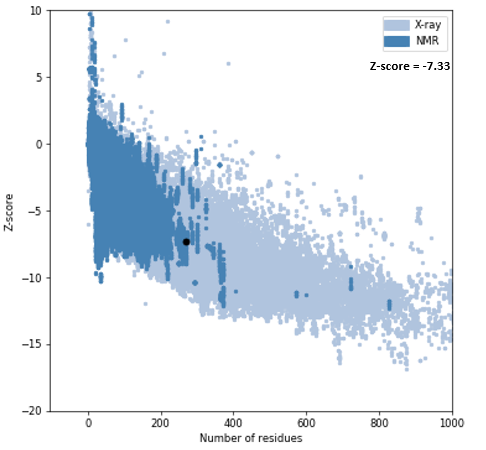

Supplement: S4 Fig — (TIF) [file pone.0273252.s004.tif]

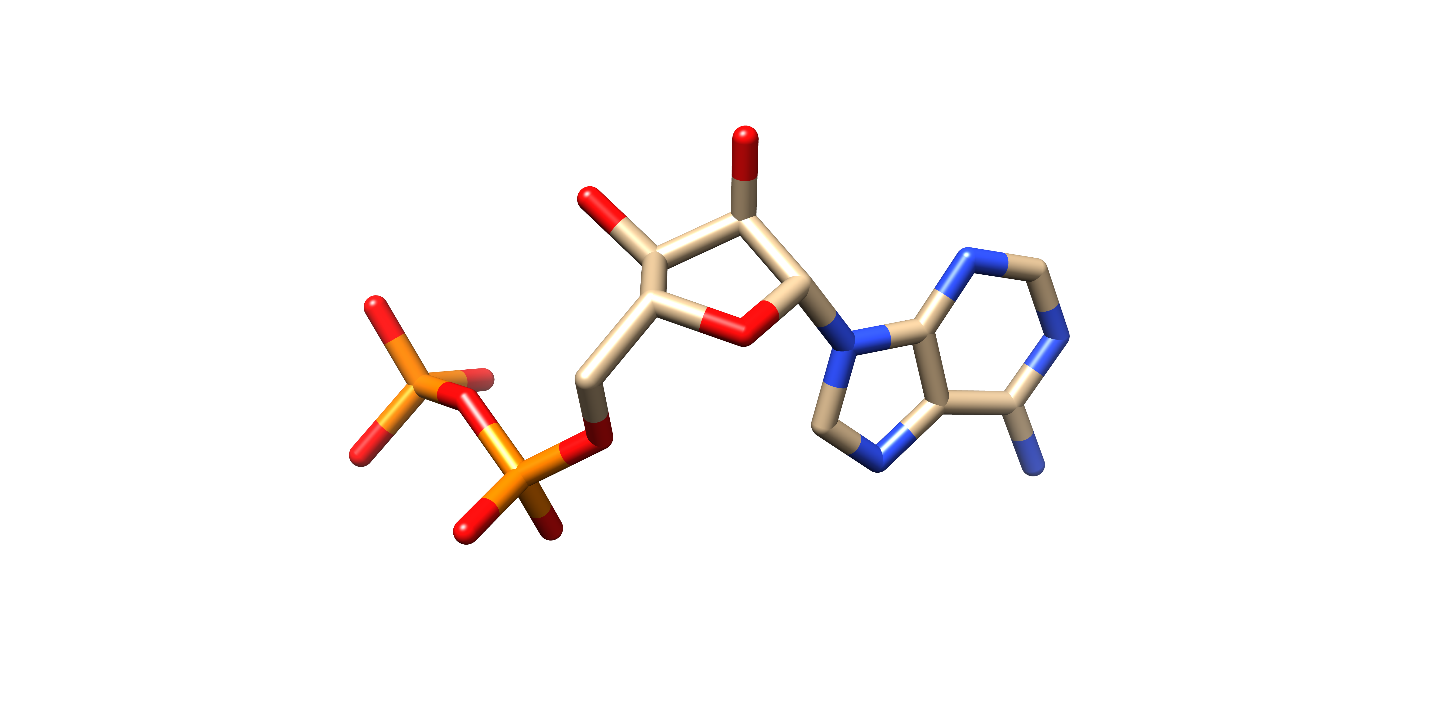

Supplement: S5 Fig — (TIF) [file pone.0273252.s005.tif]

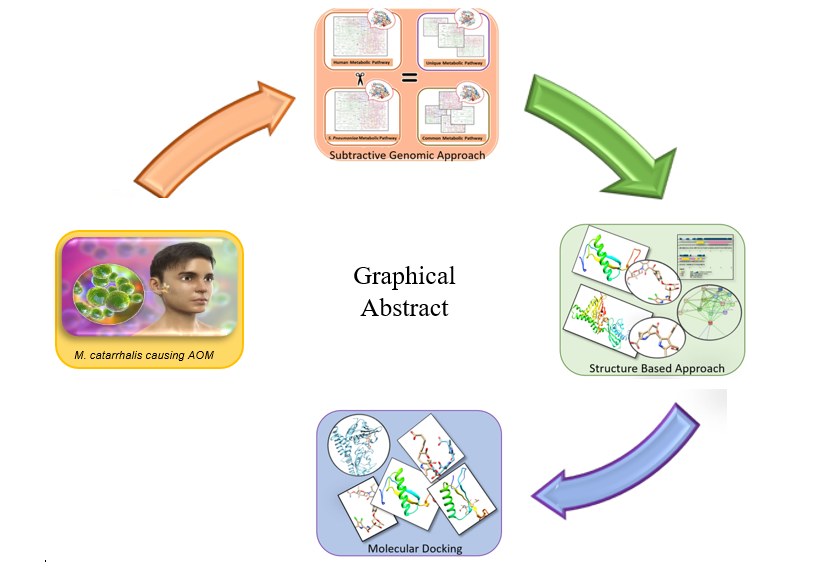

Supplement: S1 Graphical abstract — (TIF) [file pone.0273252.s006.tif]
